# Using Nutraceuticals to Help Manage Traumatic Spinal Cord Injury

**DOI:** 10.3390/ph17010071

**Published:** 2024-01-04

**Authors:** Paul Stacey, Arun Mensinkai, Pankaj Bansal, Seyed-Hossein Hosseini, Andrew Lavigne, Basia Gwardjan, Sayna Leylachian, Zhihui (Joy) Deng, Vinjamuri Chari, Sandra Giles, Shanker Nesathurai

**Affiliations:** 1 Department of Physical Medicine and Rehabilitation, Hamilton Health Sciences, Hamilton, ON L8L 2X2, Canadavchari@cogeco.ca (V.C.); 2 Division of Physical Medicine, Michael G. DeGroote School of Medicine, McMaster University, Hamilton, ON L8S 4L8, Canada; 3 Department of Diagnostic Imaging, Hamilton Health Sciences, Hamilton, ON L8L 2X2, Canada; 4 Department of Medical Imaging, Michael G. DeGroote School of Medicine, McMaster University, Hamilton, ON L8S 4L8, Canada; giles@hhsc.ca

**Keywords:** spinal cord injury, nutraceuticals, tractography, vitamin E, selenium

## Abstract

Traumatic spinal cord injury (TSCI) is a significant public health challenge that has an adverse impact on functional independence, quality of life, and life expectancy. Management of people’s chronic conditions is a key aspect of contemporary medical practice. Our study was an open label, single arm, prospective pilot study to evaluate the feasibility of treating people with TSCI. The study intervention was treatment with oral selenium and vitamin E. Participants were 18 years or older and experienced a TSCI at least one year prior to enrollment. Daily doses of 50 mcg of selenium and 400 IU of vitamin E were administered. Participants had radiologic (MRI tractography) and clinical (ASIA) assessments prior to initiating treatment, and these assessments were repeated after one year of treatment. Four subjects completed the full twelve-month study. Adherence, based on pill counts, was approximately 75% in all subjects. There were no adverse events related to study medications. During the treatment period, subjects reported improvement in certain symptoms. There was no significant difference in ASIA scores before and after the intervention. Combination treatment with vitamin E and selenium has been demonstrated as safe for TSCI patients. It is possible to use DTI values to locate the epicenter of a lesion as well as gauge the extent of injury. MRI tractography may serve as a meaningful surrogate endpoint. The results of this study suggest that it is feasible to conduct a larger long-term clinical trial to evaluate the efficacy of combination treatment of TSCI.

## 1. Introduction

Traumatic spinal cord injury (TSCI) is a significant public health challenge. The worldwide incidence is estimated to be approximately 900,000 new cases each year [1]. Due to enhanced supportive medical and surgical approaches, as well as comprehensive rehabilitation programs, people with spinal cord injury live full and productive lives. Nevertheless, there are no clearly demonstrated medical or surgical treatments to address the underlying paralysis. As people with spinal cord injury advance in age, they experience many chronic conditions. The effective management of chronic conditions in people with disabilities is a key component of contemporary medical practice. People with spinal cord injuries experience a number of chronic conditions, such as chronic pain, pressure ulcers, urinary tract infections, deep vein thrombosis, osteoporosis, depression, and respiratory complications. Some of these conditions may result in recurrent hospitalizations. Approximately 30% of persons with SCI are re-hospitalized once or more at any point following their injury, with an average rehospitalization duration of 19 to 23 days. As such, TSCIs have an adverse impact on functional independence, quality of life, and life expectancy [2]. The quest for efficacious treatments has been the focus of intense scientific endeavor. In this context, this paper presents the results of an open label, single arm, prospective pilot study of people with TSCI. The intervention was treatment with oral selenium and vitamin E. Both nutraceuticals have anti-oxidant properties. There is promising pre-clinical evidence that these two agents may be effective treatments. This pilot study also utilized magnetic resonance imaging (MRI) tractography, with is a novel method of imaging the damage and potential recovery of fiber tracts associated with TSCI. 

As a pilot study, the goal was to evaluate the feasibility of enrolling individuals with this chronic disease in a year-long trial. This included insights related to ease of subject enrollment, completion of radiological studies, adherence to treatment, as well as participation in follow-up clinical assessments. These are relevant to any future clinical trials in which adherence for extended time periods may be challenging. The goal of this pilot study was to not make inferences related to efficacy.

### 1.1. Selenium as an Antioxidant and a Potential Treatment

Selenium is an essential dietary nutrient with antioxidant properties. It is present in beef, Brazil nuts, shiitake mushrooms, and fish [3]. Selenium deficiency is associated with cardiomyopathy, myopathy, hemolysis, edematous states, and hepatic and pancreatic necrosis [4,5,6]. Supplementation with selenium may have a positive effect on a variety of diseases, including non-melanoma skin cancer, prostate cancer [7,8], type 2 diabetes [9], and autoimmune hyperthyroidism [10].

Selenium supplementation, up to 724 mcg per day, is safe. Clinical manifestations of selenium toxicity in humans are more likely with long-term intake greater than 900 mcg per day. Clinical manifestations include hair and nail loss, scalp and skin lesions, skin rash, nervous system abnormalities, vomiting, nausea, fatigue, irritability, and halitosis [11].

Selenium acts as an antioxidant by forming part of a glutathione peroxidase enzyme, a cofactor for the reduction of antioxidant enzymes. Selenium is also a scavenger of fatty acid radicals. Selenium has been tested in a cat model of SCI, and was found to improve motor function [12]. 

### 1.2. Vitamin E as an Antioxidant and Potential Treatment

Vitamin E is a fat soluble essential dietary nutrient. It is a potent antioxidant. Oral preparations of vitamin E are absorbed by the liver. Preferentially, absorption is of the D-alpha tocopherol species. Vitamin E is present in spinach, turnip greens, chard, sunflower seeds, almonds, bell peppers and asparagus. In clinical trials, 400–800 IU of vitamin E per day has reduced the incidence of cardiovascular death and nonfatal myocardial infarction in patients with atherosclerosis [13].

The upper limit for daily vitamin E intake, according to the National Academy of Sciences, is 1000 mcg per day. Potential adverse effects associated with excess intake of vitamin E include hemorrhage, fatigue, emotional disturbances, thrombophlebitis, breast soreness, creatinuria, altered serum lipid and lipoprotein levels, GI disturbances, and thyroid abnormalities [14,15].

### 1.3. Rationale for Combination Treatment

There is some evidence that selenium and vitamin E may act synergistically in SCI recovery. The rationale for combination treatment of TSCI is discussed elsewhere [16,17]. The combination of selenium and vitamin E has been demonstrated to be efficacious in a cat model of SCI. The combination treatment resulted in increased spinal cord blood flow, as well as an improvement in mean atrial pressure. Even more promising is that the combination of these two agents decreased ischemia at the level of the injury. Combination treatment also attenuated secondary injury, as evidenced by biochemical assays [18].

Selenium may attenuate oxidative stress, maintain cell membrane integrity, as well as prevent apoptosis [4,16,17,18,19,20,21,22,23]. In rats with experimental TSCI, selenium improved motor function [19]. Vitamin E may attenuate the secondary biochemical cascade. It may decrease bleeding and edema at the site of injury in rats [20,24]. It may prevent the propagation of free radicals in tissues to form a tocopherol radical [15]. Combined treatment with vitamin E has been associated with improved limb movement in cats with experimental TSCI [12,18].

In the macaque fascicularis monkey, a combination of vitamin E, selenium, and Thyrotropin-releasing-hormone (TRH), on a preliminary level, has been demonstrated as a safe treatment for TSCI [17]. TRH, as demonstrated in clinical studies, is a safe treatment for human TSCI and may result in improvement in motor function [25]. Based on overall pre-clinical and human data, the combination of TRH, vitamin E, and selenium should be considered in larger clinical trials. As a first step, the goal of this preliminary study was to explore the feasibility of a large clinical trial.

### 1.4. MRI Tractography as a Surrogate Measure of Baseline Impairment and Improvement

MRI tractography is a non-invasive method of imaging the spinal cord. It permits the measurement of fiber tracts in the spinal cord, including corticospinal fibers. MRI tractography can provide information about the size and appearance of the microstructure of nerve fiber tracts in SCI in a 3D format [26]. These tracts are involved in mediating volitional limb movement, and an increase in size is associated with an increase in speed or the number of neurons, which is associated with an improvement in volitional movement [27,28]. As such, tractography may serve a meaningful clinical role as a surrogate endpoint in evaluating treatments for TSCI.

## 2. Results

This was an open label, single arm, prospective pilot study of people with chronic TSCI. The aim of this study was to assess the feasibility of studying this group of persons with TSCI disabilities.

Initially, six potential participants were screened for enrollment. Two were deemed ineligible (one subject was taking anticoagulants, and a second had a planned surgical procedure pending). Ultimately, four subjects were enrolled. All four subjects completed the full twelve-month study.

Based on pill count data, adherence with medications was approximately 75 percent in all subjects.

Adverse events included bladder cystitis in one participant, and pneumonia in another participant. Both events resolved with oral antibiotics treatment and did not require hospitalization. These adverse events were deemed unrelated to study medications and not serious in nature.

During the treatment period, subjects reported symptoms including reduced twitching and fewer spasms, increased energy, increased sensation, fewer colds, and improved bladder function.

All four enrolled subjects had paraplegia on the basis of thoracic spinal cord injury. There was no significant difference in the American Spinal Injury Association Impairment Scale (ASIA) sensory and motor assessment before and after treatment. One subject (number 2) had a change in their ASIA sensory assessment, in which the sensory level changed from T11 to T12. ASIA motor and sensory scores are summarized in Table 1 for the four subjects. In the other three participants, baseline and final examinations were unchanged.

**Figure 1:**
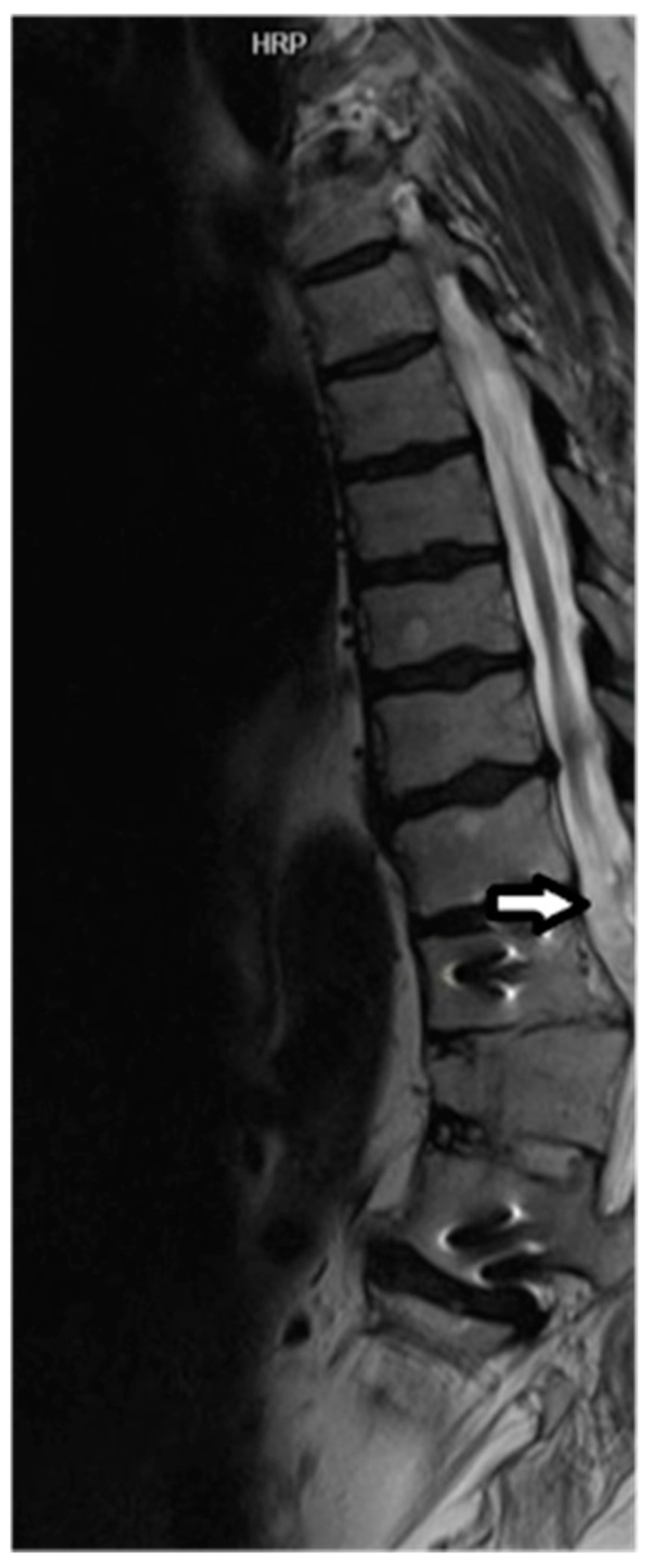
Sagittal T2 image demonstrating cord hyperintensity and volume loss proximal to the level of the TSCI (arrow). Cord T2 hyperintensity at the level of T11 vertebra corresponds to a neurological deficit found during the clinical exam. Metallic artifacts within the T11 and L1 vertebral bodies related to orthopaedic hardware from prior posterior decompression and fusion was observed. There is also a chronic anterior compression fracture of T12 noted.

**Figure 2:**
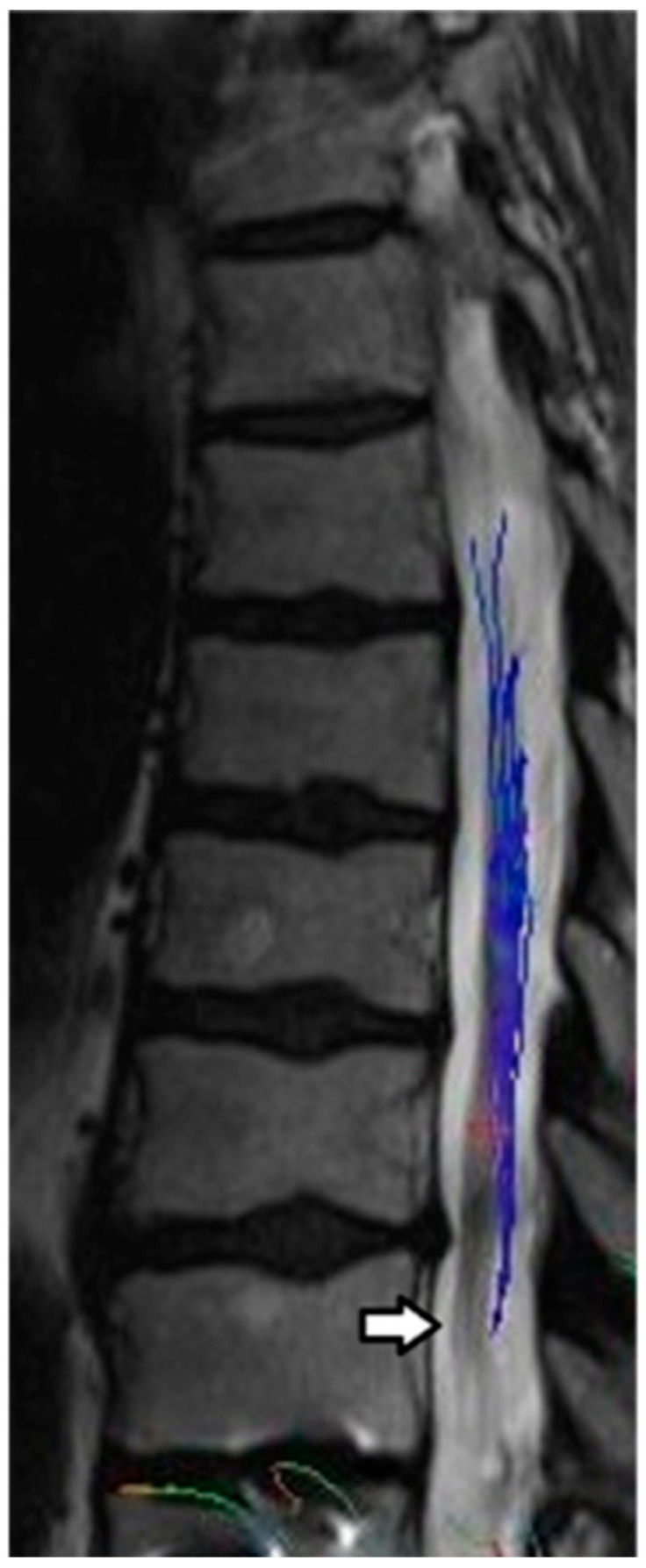
Sagittal T12 post processing MRI diffusion tractography imaging (DTI) demonstrates tapering of tract fibers in the lower thoracic cord proximal to the level of injury, corresponding to the volume loss, in keeping with Wallerian degeneration.

All four subjects were able to complete baseline and one-year MRI studies. All four subjects attended their scheduled appointments. Representative MRI images are presented in Figure 1 and Figure 2. 

## 3. Discussion

### 3.1. Feasibility of a Larger Clinical Trial

The results of this pilot study suggest that a larger clinical trial is feasible. Specifically, the investigators were able to enroll subjects with a concerted effort at one academic institution. Of note, TSCI has a relatively small pool of potential enrollees when compared to other diseases. Specifically, there are approximately 18,000 new TSCI cases in the USA each year; in contrast, there are approximately 800,000 people who experience acute coronary syndrome [2,29].

All study subjects were able to complete the baseline and follow-up MRI tractography studies.

Adherence with the study’s medications, based on pill counts, was 75 percent. As a general construct, adherence is improved with a simple treatment regimen, as well as providing medications without cost. Additionally, patient education related to the rationale for treatment, as well as regular communication, is also likely to enhance adherence. Providing pill boxes and medication reminder calls or text messages might have increased adherence. Forgetfulness is a common reason for patient non-adherence to treatment [30]. Nevertheless, from a biological perspective, the goal of the intervention was to saturate the body’s stores of these medications. In this context, missing a dose was unlikely to affect any potential efficacy.

### 3.2. Promise and Challenges of MRI Tractography

MRI tractography is a 3D modeling technique that collects data using diffusion-weighted images (DWI) or diffusion tensor images (DTI) to map structural nerve connections in the central nervous system. This imaging approach provides unique insights into the pathophysiological and microstructural alterations associated with TSCI. It provides information about neural tracts in a three-dimensional format [26]. MRI tractography is superior to conventional MRI studies with regard to identifying Wallarian degeneration.

This pilot study demonstrated that it is possible to use DTI values to locate the epicenter of a lesion, as well as gauge the extent of injury. This serves not only to support the clinical assessment, but also to guide therapy and management. Although the rostral extent of a lesion can usually be readily obtained by clinical examination, the caudal boundary is often more difficult to discern. 

There were limitations using DTI with participants. The most significant limitation was from the implanted surgical instrumentation, which resulted in image artifacts. Other sources of artifacts included cardiac and respiratory motion, as well as CSF pulsation [31]. This hampered the acquisition of data at the site of injury, as well as rostral and caudal to the lesion. Furthermore, this also affected the post-processing of data. Secondly, adequate spatial resolution remained a problem, and it was difficult to visualize the individual funiculi; this was most evident in the lower thoracic cord [32]. Moving forward, these issues can be addressed using a higher power MRI machine (i.e., a 3 Tesla magnet) as well as dedicated image-processing software.

In clinical trials, MRI tractography may serve as a meaningful surrogate endpoint. This could supplement impairment and functional endpoints. Serial MRI tractography assessments may provide insights into the underlying impairment as well as potential effects of treatment. Improvements in surrogate endpoints may translate into meaningful clinical improvement.

## 4. Materials and Methods

### 4.1. Methods

Participants were people with a remote traumatic SCI who were residing in the community. Specifically, participants must have experienced a SCI at least one year prior to enrollment and must have completed all surgical treatment(s).

### 4.2. Inclusion Criteria

Participants were 18 years or older and experienced a TSCI at least one year prior to enrollment. Other inclusion criteria included the ability to swallow pills at prescribed doses, provide informed consent, travel to the Hamilton General Hospital for radiological studies, as well as commit to attending monthly meetings with study investigators. 

### 4.3. Exclusion Criteria

Patients were excluded from participation if they had a contraindication to MRI scanning (e.g., metal, pacemaker, implanted nerve stimulator, or claustrophobia); a coexisting neurological condition (e.g., stroke, acquired brain injury, or peripheral nerve injury); a pressure ulcer at the time of enrollment; uncontrolled autonomic dysreflexia; use of anticoagulants at the time of enrollment; allergy to selenium or vitamin E; prior treatment with either nutrient; planned or anticipated surgical treatment for SCI; cauda equina or conus medullaris lesions; or known serious heart disease or diabetes.

### 4.4. Recruitment and Safety

Participants were recruited through flyers posted at Hamilton Health Sciences. Additionally, the principal investigator discussed the clinical trial with the local chapter of Spinal Cord Injury Canada, the Physical Medicine and Rehabilitation faculty, and residents at the university.

Prior to enrollment, written informed consent was obtained. Participants had twenty-four-hour access to investigators to discuss concerns. Participants were also advised of the contact information of the safety monitor, who was available to address any safety concerns. Participants were able to withdraw from the protocol at any time. The investigator met with the participants on a monthly basis. Adverse events were also reviewed with the safety monitor on a regular basis.

### 4.5. Intervention

A daily dose of 50 mcg of selenium (Natural Health Factors) was orally administrated to patients in tablet form on an empty stomach, and not with any other medications (aside from vitamin E). The medicinal ingredient of the supplement was hydrolyzed vegetable protein chelate, and non-medicinal ingredients were calcium phosphate, microcrystalline cellulose, and vegetable grade magnesium stearate.

A daily dose of 400 IU of vitamin E (Natural Health Factor) (d-alpha tocopherol) was administered orally with the selenium. The medicinal ingredient of the vitamin preparation was vitamin E, and non-medicinal ingredients included gelatin, glycerin, purified water, and soybean oil. 

These doses were consistent with over-the-counter preparations, which are available without a prescription. In this context, there was no blood level monitoring.

### 4.6. Assessment

Prior to initiating treatment, participants had radiologic and clinical assessments. The baseline clinical assessment utilized was the American Spinal Injury Association (ASIA) assessment. The baseline radiographic assessment consisted of an MRI study of the spinal cord.

The radiographic assessment (MRI study) and clinical assessment (ASIA assessment) were repeated after one year of treatment. The examiners were attending physiatrists or senior residents. A rectal exam was not completed on any subject. 

During the treatment period, study participants attended in-person monthly meetings to review any side effects and concerns related to the treatment. These meetings also served to promote compliance through pill counts and promote community for persons with chronic SCI.

## 5. Conclusions

This pilot study suggests that it is feasible to conduct a larger long-term clinical trial to evaluate the efficacy of combination treatment for TSCI. The lack of adverse effects to this particular treatment (albeit in a small study subset) provides some reassurance regarding safety. This study is relevant to any future general medical trials, as it explores the feasibility of enrolling people with a disability in a long-term trial.

## Figures and Tables

**Table 1:** ASIA assessment motor and sensory scores.

Subject	Neurological Level of Injury (Sensory, Pre-Treatment)	Neurological Level of Injury (Sensory, Post-Treatment)	Neurological Level of Injury (Motor, Pre-Treatment)	Neurological Level of Injury (Motor, Post-Treatment)
1	T10	T10	T10	T10
2	T11	T12	L4	L4
3	T5	T5	T5	T5
4	T11	T11	T11	T11

## Data Availability

Data are contained within the article.
